# How Does Digital Human Resource Management Foster a Sense of Relaxation Among Generation Z Employees?

**DOI:** 10.3390/bs16050824

**Published:** 2026-05-20

**Authors:** Hongyuan Zhang, Xin Hou, Shuming Zhao

**Affiliations:** 1 Institute of Marine Economics, Jiangsu Ocean University, Lianyungang 222005, China; 200900001@jou.edu.cn; 2 School of Business, Jiangsu Ocean University, Lianyungang 222005, China; 3 School of Business, Nanjing University, Nanjing 210093, China; zhaosm@nju.edu.cn

**Keywords:** digital human resource management, Generation Z employees, employee relaxation, work autonomy, digital self-efficacy

## Abstract

In the contemporary digital economy, digital human resource management is reshaping organizational practices and enhancing both operational efficiency and the employee experience. As Generation Z (those born between 1995 and 2009) becomes the core demographic in the workforce, their pronounced emphasis on work–life balance introduces novel managerial challenges. Drawing on conservation of resources (COR) theory, this study develops and tests a moderated mediation model examining how digital human resource management (HRM) influences sense of relaxation among Generation Z employees. Analyzing survey data from 364 Generation Z employees, we first develop and validate a measurement scale for employee relaxation, identifying four distinct dimensions: work disengagement, work adaptation, emotional regulation, and physical load. The findings reveal that digital HRM significantly enhances employee relaxation, with work autonomy serving as a partial mediator in this relationship. Furthermore, digital self-efficacy positively moderates both the direct effect of digital HRM on work autonomy and the indirect effect on employee relaxation through work autonomy. These findings offer theoretical insights into how digital HRM links to employee well-being and provide practical guidance for organizations managing a Generation Z workforce.

## 1. Introduction

Since the beginning of the 21st century, the rapid advancement of technologies such as cloud computing, big data, and artificial intelligence has driven the penetration of the digital economy into all areas of economic and social life (X. Liu, 2025). This digital transformation has fundamentally reshaped organizational processes and business models (Vial, 2021), while also creating new opportunities and challenges for the digital workforce (Colbert et al., 2016). However, it also raises concerns about job insecurity and the need for technical skill upgrades (Arranz Lahuerta et al., 2026). Within this context, human resource management (HRM), as a critical component of organizational management, is undergoing a profound digital transformation, leading to the emergence of digital HRM (Y. P. Li et al., 2021). Research indicates that digital HRM not only serves as a communication bridge between employees and organizations but also acts as a tool for management to promptly capture fluctuations in employee psychological states (Ding, 2024; Van De Voorde et al., 2012). However, existing studies have predominantly focused on the impact of specific digital technologies on employee behavior and attitudes. A growing body of research has also highlighted the negative consequences of digitalization, such as technostress, a phenomenon where employees experience stress due to the use of information technology (Ayyagari et al., 2011; Srivastava et al., 2015). Conversely, some studies have shown that human–robot collaboration can lead to positive behavioral changes in service contexts (Wu et al., 2025b). There remains a lack of sufficient empirical examination regarding the practical outcomes of digital HRM, formed through the deep integration of digital technology and HRM, particularly its mechanisms of action on employee attitudes and behaviors (Z. Liu et al., 2023). Concurrently, Generation Z employees (born between 1995 and 2009) are entering the workforce en masse and gradually becoming the dominant cohort. Their developmental trajectory has been seamlessly intertwined with the evolution of internet technology (Ao, 2021), resulting in distinctly pronounced generational characteristics (Gao & Huang, 2024). The research has documented generational differences in work values and attitudes, with Generation Z showing stronger preferences for leisure, autonomy, and work–life balance compared to previous generations (Twenge et al., 2010; Lyons et al., 2015). Data from the “2023 Workplace Generational Insights” report released by 51job indicates that the turnover rate among Generation Z employees is significantly higher than that of other generational groups, with 40% of post-2000s employees citing high work pressure as the primary reason for leaving. Furthermore, Shi and Li (2024) note that Generation Z employees have heightened expectations for their work environment, pursuing comfortable, autonomous, and flexible work arrangements that emphasize work–life balance (Shi & Li, 2024). This suggests that, compared to preceding generations, Generation Z has a stronger and more urgent need for a sense of relaxation in the workplace. Currently, a unified academic definition of employee relaxation remains absent. In this study, we define employee relaxation as a perceived psychological state characterized by low levels of tension, the absence of work-related rumination during off-job time, and a subjective sense of ease and comfort in the workplace, and low levels of perceived physical tension and fatigue. This definition has three key features. First, it emphasizes perceived state rather than stable traits, capturing momentary workplace experiences. Second, it focuses on the absence of strain rather than merely the presence of positive affect. Third, it distinguishes relaxation from general well-being: while well-being encompasses life satisfaction and positive emotions broadly, relaxation specifically captures the workplace-specific experience of being at ease and free from pressure. This state is posited to help employees mitigate excessive stress, foster creativity and innovative thinking, and enhance both focus and efficiency. In the era of the digital economy, as digital HRM becomes a central element of organizational management, understanding how it influences the sense of relaxation among Generation Z employees constitutes a critical and timely research imperative. While digital HRM has attracted considerable research attention, less is known about how it affects employees’ psychological states, particularly the sense of relaxation that has become increasingly important for Generation Z employees. This study focuses on this question.

Although current research has not directly examined the relationship between digital human resource management (HRM) and relaxation among Generation Z employees, existing studies provide a foundation for clarifying this linkage. At the theoretical level, conservation of resources (COR) theory suggests that resources—defined as personal characteristics, energies, or conditions that individuals value—are central to understanding workplace dynamics, and that fluctuations in work resources significantly influence individuals’ emotions and behaviors (Hobfoll, 2001). Digital HRM, enabled by information and communication technologies, can be conceptualized as a work resource (B. Wang et al., 2020). By leveraging digital and intelligent technologies to execute HR functions such as recruitment, training, and administration, digital HRM provides Generation Z employees with enriched learning opportunities and job resources, thereby alleviating the burden of job demands (Bakker & Demerouti, 2007), facilitating the formation of relaxed states, and ultimately enhancing relaxation. At a logical level, digital HRM offers greater flexibility through practices such as remote work and flexible scheduling, enabling Generation Z employees to autonomously arrange their work pace and enhancing their sense of control over work timing and location. This, in turn, fosters work autonomy. Enhanced work autonomy reduces constraints from rigid work patterns, thereby strengthening Generation Z employees’ sense of relaxation. These considerations suggest that digital HRM may not only exert a direct effect on the relaxation of Generation Z employees but also indirectly influence it through the mediating mechanism of work autonomy.

It is noteworthy that employees often exhibit heterogeneous mindsets when confronted with new technological tools. Xu et al. (2025) found that human–AI collaboration may lead to unintended negative behaviors, such as cyberloafing, depending on employees’ AI identity perceptions (Xu et al., 2025). Digital self-efficacy, as a dynamic psychological variable, reflects an individual’s perception of and confidence in their ability to utilize digital devices (Ulfert-Blank & Schmidt, 2022). Employees with high digital self-efficacy typically possess stronger technological adaptability, enabling them to acquire new skills and tools more rapidly and to thrive in digital work environments. Conversely, employees with low digital self-efficacy often lack confidence in their digital capabilities, tend to rely on others to complete digital tasks, and may consequently experience diminished work autonomy. Therefore, this study introduces digital self-efficacy as a moderating variable to explore its contingent role in the process through which digital HRM influences relaxation among Generation Z employees.

Applying COR theory to the context of digital HRM and Generation Z employees offers unique theoretical value for three reasons. First, digital HRM represents a novel form of organizational resource that differs from traditional HRM practices. Unlike conventional HRM, which often operates through fixed policies and face-to-face interactions, digital HRM provides employees with anytime-anywhere access to work resources. This unique feature makes digital HRM particularly suitable for triggering resource gain spirals, as employees can continuously access and mobilize resources without temporal or spatial constraints. Second, Generation Z employees exhibit distinct resource dynamics compared to previous generations. Growing up with digital technologies, Generation Z employees have high digital literacy but also high expectations for workplace flexibility and autonomy. From a COR perspective, they are more sensitive to resource threats and more responsive to resource gains that enable autonomy and self-determination. This generational difference suggests that the mechanisms linking organizational resources to employee outcomes may operate differently for Generation Z, offering a boundary condition for COR theory. Third, the interaction between digital HRM and Generation Z creates a natural laboratory for examining resource gain spirals. The rapid digitization of HRM coincides with Generation Z’s entry into the workforce, allowing us to observe how a generation with high digital efficacy responds to digitally enabled work resources. This context provides an opportunity to test COR theory’s propositions about resource caravans and gain spirals in a contemporary setting.

Based on the foregoing analysis, this study focuses on employed Generation Z individuals and draws upon COR theory to investigate the mechanisms by which digital HRM affects their sense of relaxation. Specifically, this study aims to address the following questions: (1) Does digital HRM positively influence Generation Z employees’ sense of relaxation? (2) Does work autonomy mediate this relationship? (3) Does digital self-efficacy moderate the indirect effect of digital HRM on relaxation through work autonomy? By addressing these questions, this research seeks to reveal the underlying mechanisms through which digital HRM shapes the relaxation of Generation Z employees, thereby offering a theoretical foundation and practical insights for organizations seeking to optimize their management of the Generation Z workforce.

## 2. Theoretical Basis and Research Hypothesis

### 2.1. Digital Human Resource Management and Employee Relaxation

Conservation of Resources (COR) theory (Hobfoll, 2001) posits that individuals strive to obtain, protect, and maintain resources that they value. Subsequent work has further elaborated the role of resources in organizational settings, emphasizing the dynamic nature of resource gain and loss spirals (Halbesleben et al., 2014; Hobfoll et al., 2018). Resources can be objects, conditions, personal characteristics, or energies. The theory identifies two core mechanisms that explain how resources affect employee outcomes. The first mechanism is the resource loss spiral: when individuals experience resource depletion, they become more vulnerable to further losses, leading to negative outcomes such as stress and burnout. The second mechanism is the resource gain spiral: initial resource gains can generate further gains, creating upward spirals that enhance well-being and positive outcomes. In this study, we focus on the resource gain spiral mechanism. Digital HRM functions as an initial resource gain by providing Generation Z employees with flexible work arrangements, real-time information access, and reduced administrative burdens. According to COR theory, this initial resource gain should trigger a positive spiral: employees who receive digital HRM resources are better able to acquire additional resources, which in turn fosters a state of relaxation. We elaborate this mechanism in the following hypotheses.

Digital HRM refers to the practice of using digital technology to collect, analyze, and apply human resource data, constructing a new operational model through data-driven decision-making. It is characterized by precision, convenience, and customization (Y. P. Li et al., 2021), and its essence lies in empowering HRM practices with digital technology to optimize the process of HRM. Digital HRM breaks the constraints of traditional management models, creating a more flexible and efficient working environment for employees. With the help of digital platforms such as Yammer, DingTalk, and Mingdao, remote work and flexible working arrangements can be effectively implemented (A. M. Li et al., 2024). Employees can independently arrange their work locations and schedules according to their personal needs, better achieving a balance between work and life, thereby reducing work pressure and fatigue (B. C. Wang et al., 2025). Compared to other generations of employees, Generation Z, who have grown up in an environment where digital technology has developed rapidly, have a higher acceptance and stronger demand for digital HRM. They are more accustomed to using digital tools to solve problems, more resistant to high-pressure and controlling management styles, and show a clear preference for the flexibility and freedom provided by digital management. Under this management model, Generation Z employees can reduce stress from both physical and mental aspects and achieve a higher degree of relaxation.

**Hypothesis** **1:** *Digital human resource management positively promotes relaxation among Generation Z employees*.

### 2.2. The Mediating Role of Work Autonomy

The concept of work autonomy initially originated from the job characteristics model proposed by Hackman and Oldham, referring to the degree of autonomy employees have in arranging work hours, determining work progress, and deciding on work processes (Hackman & Oldham, 1975). From a motivational perspective, work autonomy is a core psychological need that enhances intrinsic motivation and well-being (Gagné & Deci, 2005). Contemporary research has also highlighted the critical role of job design in promoting employee health and performance (Parker, 2014). With the rapid development of digital technologies such as big data and artificial intelligence, the working styles of Generation Z employees have undergone profound changes, promoting their labor independence and work autonomy. Research indicates that the work autonomy of Generation Z employees encompasses labor management autonomy, including autonomy in ideological pursuit, career choice, and technology application (Jian, 2024).

As the “first generation to grow up entirely in the digital age”, they excel at expressing personal views, prioritize subjective feelings and self-awareness, and have developed independent thinking and diversified values. Taking the cultural and creative industry as an example, over half of its employees are under the age of 35, making it a field heavily populated by Generation Z employees. The cultural and creative industry relies heavily on creativity and innovation, and its development cannot be separated from practitioners who possess autonomy and initiative (Chen & Liang, 2024). In the context of digitalization and intelligence, digital human resource management creates an environment for Generation Z employees to freely express their views and make independent decisions by providing an open information platform and diverse communication channels. With the help of digital and intelligent technologies, they can fully unleash their boundless creativity, seek inspiration, and drive the development of the cultural and creative industry, thereby strengthening the ideological pursuit of autonomy among Generation Z employees. Secondly, digital human resource management helps Generation Z employees explore their career paths independently, enhance their skills, and make more informed career choices based on their personal interests and goals by providing personalized career planning tools, implementing performance feedback, and flexible work arrangements, thus enhancing their autonomy in career selection. Lastly, digital human resource management provides extensive digital and intelligent technical support for Generation Z employees, thereby providing technical support for enhancing their work autonomy (Jian, 2024), enhancing their sense of technological acquisition, and promoting their autonomy in technology application. It can be seen that digital human resource management significantly enhances the ideological pursuit of autonomy, career choice autonomy, and technology application autonomy of Generation Z employees by providing an open information platform, flexible work arrangements, and advanced digital technologies.

**Hypothesis** **2:** *Digital human resource management has a positive promoting effect on the work autonomy of Generation Z employees*.

According to the Conservation of Resources (COR) theory, work autonomy, as an important job resource, can endow employees with stronger control and flexibility over their work (Bakker & Demerouti, 2007). High work autonomy enables employees to independently allocate work resources, effectively reducing feelings of constraint and enhancing control over the work process. Research indicates that work autonomy can significantly alleviate work pressure by providing more freedom and choice (Lopes et al., 2014). As the new generation in China, Generation Z pursues individual autonomy in the online digital realm of the Internet, gradually forming a personalized lifestyle that satisfies self, advocates happiness, and pursues freedom (Y. Wang & Fan, 2024). High work autonomy is highly consistent with their inherent need for self-control and flexible work arrangements, thereby helping to reduce work pressure and enhance job relaxation.

According to the Conservation of Resources (COR) theory, individuals have a fundamental tendency to preserve, acquire, and compensate for resources, and a state of resource balance can bring satisfaction and happiness (Hobfoll, 2001). Digital human resource management creates valuable work resources for Generation Z employees by providing abundant training opportunities, open information platforms, and diverse communication channels. These resources not only enhance employees’ skills and knowledge levels but also open up a larger space for autonomous work. In such an environment, Generation Z employees can more flexibly arrange work tasks and independently choose suitable work methods, thereby significantly enhancing work autonomy. As an external work resource, work autonomy can effectively alleviate work pressure, compensate for resource depletion, increase employees’ psychological resource reserves, and ultimately enhance job and life satisfaction (Fei et al., 2017). This process precisely meets the needs of Generation Z employees for self-actualization and personalized development, allowing them to experience a stronger sense of control and achievement, ultimately enhancing job relaxation.

**Hypothesis** **3:** *Work autonomy mediates the relationship between digital human resource management and Generation Z employees’ sense of relaxation*.

### 2.3. The Moderating Effect of Digital Self-Efficacy

Digital self-efficacy stems from the extension of the concept of self-efficacy by scholars such as Agarwal. It primarily refers to an individual’s confidence in their ability to use information technology and adapt to updates in digital devices, and is an individual’s subjective perception of completing tasks related to digital systems (Agarwal et al., 2000). Unlike self-efficacy, which emphasizes comprehensive abilities, digital self-efficacy focuses more on specific ability perceptions in the context of digital organization (Ulfert-Blank & Schmidt, 2022).

In the digital context, different individuals exhibit differences in self-efficacy and other aspects (Zhang et al., 2018). Individuals with high digital self-efficacy are able to adjust resource allocation and behavior based on the information they perceive (Zhu & Zhang, 2024), and they usually have higher expectations for outcomes. Therefore, when faced with work tasks and challenges, they are more inclined to proactively utilize the digital tools provided by digital human resource management to take action, thereby significantly enhancing work autonomy. Especially for Generation Z employees who have grown up with digital technology, they generally possess strong adaptability and usage abilities of digital tools. In the digital human resource management environment, they often exhibit a higher level of digital self-efficacy, are able to flexibly respond to work challenges, effectively utilize digital tools to improve work efficiency, and further enhance work autonomy.

**Hypothesis** **4:** *Digital self-efficacy plays a positive moderating role between digital human resource management and work autonomy, meaning that the higher the digital self-efficacy, the stronger the positive impact of digital human resource management on work autonomy*.

Digital human resource management creates fundamental conditions for enhancing employees’ work autonomy by providing a rich array of digital tools and resources, such as information sharing platforms and online training. For Generation Z employees, when their digital self-efficacy is at a high level, they can adapt to and proficiently use various digital tools more quickly, thereby arranging work tasks more effectively, selecting suitable work methods, and significantly enhancing work autonomy. This increase in autonomy not only strengthens employees’ sense of control in the digital environment but also helps them better cope with work challenges, thus promoting the formation of job slack. Conversely, when digital self-efficacy is low, employees lack confidence in using digital tools, work autonomy is limited, and the promotion of job slack is correspondingly weakened.

**Hypothesis** **5:** *Digital self-efficacy positively moderates the mediating effect of work autonomy on the relaxation of Generation Z in digital human resource management. That is, the stronger the digital self-efficacy, the stronger the indirect influence of digital human resource management on the relaxation of Generation Z employees through work autonomy*.

**Figure 1:**
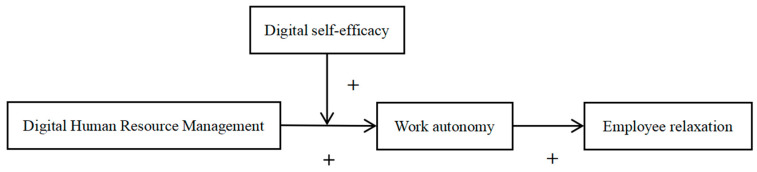
Theoretical research model (made by authors, 2025). Note: “+” indicates a positive effect.

In summary, the hypothetical model of this study is illustrated in Figure 1:

## 3. Development of the Employee Relaxation Scale

### 3.1. Data Selection and Collection

This study commenced with a systematic search of literature related to “relaxation”, “employee well-being”, and related topics in both Chinese and English databases to comprehensively understand the research progress in this field. Building upon this foundation, semi-structured interviews were employed to collect primary qualitative data. In accordance with the requirements of social statistics for sample representativeness, a total of 30 employed individuals from diverse industries, including internet and finance, were selected as interview participants. Their ages ranged from 25 to 52 years, their educational backgrounds were predominantly at the bachelor’s and master’s levels, and their positions encompassed ordinary employees, middle managers, and senior managers, thereby ensuring the diversity and representativeness of the sample. It should be noted that this study followed the classic two-stage scale development design (Churchill, 1979; DeVellis & Thorpe, 2021). The first stage included employees aged 25–52 to capture diverse relaxation experiences and ensure the dimensions were not specific to Generation Z. The second stage specifically targeted Generation Z employees to validate the scale. This two-stage design balances content validity and target population specificity. Prior to each interview, the researchers explained the purpose and content of the study to the participants. During the interviews, the researchers actively guided the conversation, encouraging participants to express their views authentically, thereby obtaining rich qualitative material. All interview content was audio-recorded and transcribed verbatim, ultimately yielding 30 valid interview transcripts. Of these, 25 transcripts were used for grounded theory coding analysis, while the remaining 5 were reserved for theoretical saturation testing.

### 3.2. Data Analysis

Grounded theory, as a bottom-up qualitative research method, primarily involves the systematic induction of concepts, categories, and theoretical frameworks from raw data (Glaser et al., 1968). This study adopts this approach, drawing upon the scale development procedures outlined by Sun et al. (Sun et al., 2020) to systematically analyze and review the collected qualitative textual data through sequential stages of open coding, axial coding, and selective coding. During the open coding phase, through in-depth analysis of the core textual materials, over 350 original statements and corresponding concepts were extracted. These were subsequently refined into 35 initial categories, with careful attention paid to preserving the authenticity of the original data and ensuring the natural emergence of concepts. In the subsequent axial coding phase, the initial categories were screened, merged, and clustered based on conceptual relationships, resulting in the formation of 16 subcategories. Finally, during the selective coding phase, integrating the inherent logical relationships among these subcategories, four overarching main categories were identified: “work disengagement”, “work adaptation”, “emotional regulation”, and “physical load” (see Table 1). Within this framework, the “work disengagement” dimension refers to employees’ ability to achieve an effective balance between work and personal life, reasonably allocating time, energy, and resources, thereby enhancing individual physical and mental well-being as well as organizational sustainability. The “work adaptation” dimension captures employees’ psychological sense of environmental ease, the internal perception of feeling comfortable, accepted, and unthreatened in the workplace. It measures the subjective perceptual outcome of external resources (e.g., support, autonomy), not the objective presence of those resources. Thus, it represents a component of the relaxation state itself, not an antecedent. Notably, work adaptation is distinct from work autonomy: work autonomy serves as a mediating antecedent, whereas work adaptation is a core dimension of employee relaxation. The “emotional regulation” dimension reflects employees’ capacity to maintain a natural, relaxed, and positive psychological state at work. The “physical load” dimension captures the physiological impact of high-intensity work on employees’ physical condition, as well as their engagement in proactive recovery behaviors. It should be emphasized that dimensions such as work disengagement and physical load are not inherently negative or pathological. Rather, they reflect the absence of excessive work-related strain. Work disengagement captures the ability to psychologically detach from work during non-work hours, a key resource recovery process that prevents burnout (Sonnentag & Fritz, 2015). Meta-analytic evidence confirms that psychological detachment from work is a crucial recovery experience that predicts well-being and reduces burnout (Bennett et al., 2018; Wendsche & Lohmann-Haislah, 2017). Early studies also established that switching off mentally from work during leisure time is essential for maintaining health (Sonnentag & Bayer, 2005). Physical load reflects the perceived level of bodily tension, where lower scores indicate relaxation and physical ease. Thus, all four dimensions align with the conceptualization of relaxation as a low-strain positive state, rather than a state characterized by high activation or positive affect.

To assess theoretical saturation, this study conducted an independent analysis using the five reserved interview samples. Two researchers who had not participated in the earlier coding stages independently analyzed these reserved materials, following the same grounded theory procedures employed in the main analysis. This process revealed no new dimensional relationships. Additionally, three experts in the field of human resource management were invited to review and evaluate the established four-dimensional structure. The results confirmed the absence of new concepts or relationships, indicating that the four-dimensional structure had reached theoretical saturation.

### 3.3. Scale Structure Examination

#### 3.3.1. Exploratory Factor Analysis

This study distributed 364 electronic questionnaires through a third-party platform. After screening the collected questionnaires, 315 valid samples were obtained. The distribution of samples is as follows: 51% are male; 39% are aged 30 and below, and 34% are aged 30–40; 80% have bachelor’s degree or below, and 20% have master’s degree or above; 30% have worked for 3 years or less, 13% for 3–5 years, and 57% for more than 5 years; 29.8% of the positions are in research and development, and 27.9% are in technology; 27.3% of the industries are in manufacturing, and 15.5% are in finance.

In terms of exploratory factor analysis, we first subjected the scale to KMO and Bartlett’s test. The KMO value was found to be 0.908, with a Bartlett’s test result of 3524.334, df = 120, and *p* = 0.000 (*p* < 0.001). Based on this result, it was indicated that the data was suitable for factor analysis. Furthermore, principal component analysis was employed, and factors were extracted according to the principle of eigenvalues greater than 1. Varimax orthogonal rotation was used to obtain the cumulative variance contribution rate of the factors and the factor load values for each item. At the same time, according to the criteria proposed by Churchill (1979), items with factor load less than 0.5 or cross-load exceeding 0.4 were deleted. Based on the analysis results, two items B5 and C1 that did not meet the load criteria were deleted. The exploratory factor analysis results are shown in Table 2. Four factors with characteristic roots greater than 1 were extracted, with eigenvalues of 7.566, 2.173, 1.233, and 1.014, respectively. The cumulative variance explained by the four factors was 74.913%, indicating that the factor analysis results were ideal.

#### 3.3.2. Confirmatory Factor Analysis

To test the fit of each factor in the employee relaxation scale, we distributed another 382 electronic questionnaires. After screening the questionnaires, we obtained 341 valid samples. The sample characteristics are as follows: 57% are male; 42% are aged 30 and below, and 33% are aged 30–40; 55% have a bachelor’s degree, and 5% have a master’s degree or above; 29% have 3 years or less of work experience, and 27% have 3–5 years of work experience; 25.5% of the positions are in research and development, and 29.6% are in technical roles; 29% are in manufacturing, and 19.4% are in the financial industry.

Using AMOS 29 software, we conducted a confirmatory factor analysis on 14 items. The results are presented in Table 3, where the four-factor model demonstrates the best fit: $\chi^{2}/df$ = 1.951, GFI = 0.945, CFI = 0.972, NFI = 0.945, TLI = 0.945, RMSEA = 0.053, SRMR = 0.042. All fit indices meet the required standards, indicating that the employee relaxation scale obtained through confirmatory factor analysis possesses good construct validity.

#### 3.3.3. Reliability and Validity Testing

The reliability test primarily involves combining the Cronbach’s α coefficients of the overall scale and each dimension. The Cronbach’s α coefficient for the overall employee relaxation scale is 0.901; the Cronbach’s α coefficients for work disengagement, work adaptation, emotional regulation, and physical load analysis and prediction are 0.828, 0.870, 0.868, and 0.770, respectively. The Cronbach’s α coefficients for both the overall scale and each dimension are above 0.7, indicating that the employee relaxation scale developed in this study has good reliability.

The validity indicators in this study mainly include convergent validity and discriminant validity. In terms of convergent validity, the values of Composite Reliability (CR) and Average Variance Extracted (AVE) are primarily used to assess the convergent validity of the scale. The AVE values for the four factors range from 0.53 to 0.688, all of which are greater than 0.5; the CR values range from 0.771 to 0.8, all of which are greater than 0.7, indicating that the dimensions of the employee relaxation scale exhibit satisfactory convergent validity. In terms of discriminant validity, the main criterion is whether the square root of the AVE value for a variable (the diagonal number in Table 4) is greater than the correlation coefficient between that variable and other variables (the numbers in the lower triangular region below the diagonal). According to the results presented in Table 4, the measurement model of this study demonstrates good discriminant validity.

## 4. Research Design

### 4.1. Research Subjects and Procedures

This study takes Z-generation employees born after 1995 as the research subjects, collects data through online questionnaires, and covers representative industries such as the Internet, finance, and technology. To mitigate common method bias, we employed a two-stage survey design: the first stage measures digital human resource management, digital self-efficacy, and control variables, with a total of 456 questionnaires collected and 418 valid questionnaires; the second stage survey is conducted two weeks later, targeting the valid respondents from the first stage to measure employee relaxation and work autonomy, with 403 questionnaires collected and 364 valid questionnaires. The demographic characteristics of the valid samples are as follows: in terms of gender, males account for 51% and females account for 49%; in terms of age, those born after 1995 account for 56% and those born after 2000 account for 44%; in terms of education, master’s degree or above account for 26.3%, bachelor’s degree account for 58%, and associate degree or below account for 15.7%; in terms of work tenure, those with 1–3 years account for 37.6%; in terms of enterprise nature, state-owned enterprises account for 14.3%.

### 4.2. Measurement Tools

1. Digital Human Resource Management: Utilizing the 10-item scale designed by Hu and Chen (2024), the items encompass “I perceive that my enterprise has platform-based management of human resource-related documents” and “I perceive that my enterprise conducts talent inventory through the human resource office platform”, with 1 indicating “completely disagree” and 7 indicating “completely agree”. In this study, the Cronbach’s α of this scale is 0.909.
2. Work autonomy: A 7-item scale developed by foreign scholars Kirmeyer and Shirom (1986) was adopted, with items including “I am free to decide what to do during work hours” and “I am free to decide how my work should be done”. The scale ranges from 1 (strongly disagree) to 7 (strongly agree). In this study, the Cronbach’s α of this scale is 0.938.
3. Digital self-efficacy: The measurement scale developed by Kim (2021) was adopted, consisting of 3 items, including “I think I can easily learn how to use digital devices” and “I believe I can use digital devices well”. The scale ranges from 1 (strongly disagree) to 7 (strongly agree). In this study, the Cronbach’s α of this scale was 0.904.
4. Employee relaxation: The scale developed in this study was used. In this study, the Cronbach’s α of this scale was 0.971.
5. Control variables: Gender, age, education level, work experience, and enterprise nature are considered as basic factors predicting employee behavior and can be effectively measured, so they are taken as control variables.

## 5. Data Analysis and Hypothesis Testing

### 5.1. Reliability and Validity Testing

All Cronbach’s α values exceeded 0.7, indicating high reliability. The factor load values for each item were all greater than 0.5, the average variance extraction values were all greater than 0.5, and the composite reliability values were all above 0.6, indicating high convergent validity. The goodness of fit for different models was obtained through confirmatory factor analysis, and the results are shown in Table 5. The four-factor model exhibited the best fit ($\chi^{2}$ = 991.5, df = 521, CFI = 0.953, TLI = 0.950, RMSEA = 0.05, SRMR = 0.041), thus indicating high discriminant validity among variables.

### 5.2. Common Method Bias Test

Due to the fact that this study primarily relied on anonymous self-assessment by employees during the survey, to prevent common method bias issues, it first employed Harman’s single-factor method to test for common method bias. The results indicated the presence of four factors with eigenvalues greater than 1, and the first factor accounted for 38% of the variance, which is below the critical threshold of 40%. This preliminarily suggests that there is no significant common method bias issue. In addition to Harman’s single-factor test, we employed the unmeasured latent marker variable approach (Podsakoff et al., 2012), a more robust method for detecting common method bias. Specifically, we introduced a latent common method factor and compared the model fit with the original four-factor model. The results showed good fit indices (CFI = 0.969, TLI = 0.964, RMSEA = 0.042, SRMR = 0.0338). Compared to the four-factor model, both CFI and TLI showed an improvement of less than 0.1, while RMSEA and SRMR showed a decrease of less than 0.05, indicating that common method bias is not a serious concern in this study (Wen et al., 2018).

### 5.3. Descriptive Statistics and Correlation Analysis

The mean, standard deviation, and correlation coefficients between variables are presented in Table 6. Digital human resource management exhibits a significant positive correlation with work autonomy (r = 0.27, *p* < 0.01) and employee relaxation (r = 0.32, *p* < 0.01). Similarly, work autonomy shows a significant positive correlation with employee relaxation (r = 0.38, *p* < 0.01). The results of the correlation analysis preliminarily align with the direction of this study’s hypothesis, laying the foundation for the subsequent hypothesis testing.

### 5.4. Hypothesis Testing

#### 5.4.1. Main Effect and Mediation Effect Tests

The hypothesis was further validated through hierarchical regression analysis, and the results are presented in Table 7. As shown in Model M6, digital HRM exerts a significant positive direct effect on employee relaxation of Generation Z employees (β = 0.312, *p* < 0.001), thus verifying Hypothesis H1. According to Model M2, digital human resource management promotes work autonomy among Generation Z employees (β = 0.365, *p* < 0.001), thus verifying Hypothesis H2. Model M7 incorporates both digital human resource management and work autonomy into the regression equation. The results indicate that work autonomy positively predicts Generation Z employees’ sense of relaxation. (β = 0.314, *p* < 0.01), but the main effect of promotion is significantly reduced (β = 0.197, *p* < 0.01). This indicates that work autonomy partially mediates the relationship between digital human resource management and the relaxation of Generation Z employees, with a positive mediating effect, thus verifying Hypothesis H3. To ensure the robustness of the results, a Bootstrap analysis was further conducted to test the mediating effect of work autonomy, with a repeated sampling size set at 5000. The results show that the total effect of digital human resource management on the relaxation of Generation Z employees through work autonomy is 0.422, with a 95% confidence interval (CI) of [0.287, 0.556]; the direct effect is 0.266, with a 95% CI of [0.129, 0.403]; and the indirect effect is 0.155, with a 95% CI of [0.088, 0.231], all of which exclude 0 from their confidence intervals. This result indicates that work autonomy plays a mediating role between digital human resource management and the relaxation of Generation Z employees, thus verifying H3 again.

#### 5.4.2. Moderating Effect Test

**Figure 2:**
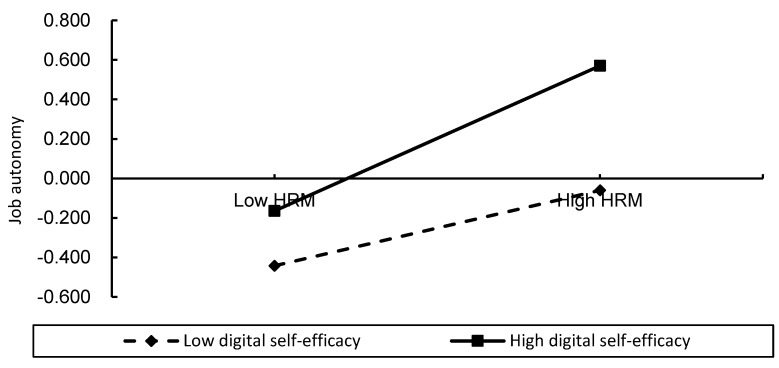
Moderation effect diagram (made by authors, 2023).

According to the conventional testing method for moderating effects, digital human resource management, work autonomy, and digital self-efficacy were standardized, and interaction terms were constructed. Hierarchical regression results showed that digital human resource management significantly positively influenced work autonomy (M3, β = 0.303, *p* < 0.01), and digital self-efficacy positively influenced work autonomy (M3, β = 0.233, *p* < 0.01). Based on M3, when the interaction term between digital human resource management and digital self-efficacy was added, the coefficient of the interaction term was significantly positive (M4, β = 0.106, *p* < 0.05), indicating that digital self-efficacy played a positive moderating role between digital human resource management and work autonomy. That is, the stronger the digital self-efficacy, the greater the enhancement effect of digital human resource management on work autonomy. Hypothesis H4 was verified, and the moderating effect is shown in Figure 2.

#### 5.4.3. Moderated Mediation Effect Test

Using the bootstrap method (with 5000 samples), we tested the indirect effect of digital human resource management on the relaxation of Generation Z employees through work autonomy, under varying levels of digital self-efficacy. The results are presented in Table 8. At a high level of digital self-efficacy, the indirect effect of digital human resource management on the relaxation of Generation Z employees through work autonomy is 0.160, with a 95% confidence interval (CI) of [0.086, 0.242]. At a low level of digital self-efficacy, the indirect effect is 0.079, with a 95% CI of [0.021, 0.149]. The difference in indirect effects between the two levels is 0.119, with a 95% CI of [0.064, 0.184], which does not include 0. Therefore, hypothesis H5 is supported.

## 6. Research Conclusions and Recommendations

### 6.1. Discussion and Conclusions

Following a systematic scale development procedure, this study identifies four dimensions of employee relaxation: work disengagement, work adaptation, emotional regulation, and physical load. Drawing on conservation of resources theory and focusing on Generation Z employees as the research population, this study empirically investigates how digital human resource management (HRM) influences employee relaxation. The key findings are as follows: digital HRM exerts a significant positive effect on employee relaxation; work autonomy partially mediates this relationship; and digital self-efficacy positively moderates the impact of digital HRM on work autonomy.

### 6.2. Discussion of Findings

First, the four-dimensional structure of employee relaxation. Our finding that employee relaxation comprises work disengagement, work adaptation, emotional regulation, and physical load extends previous conceptualizations of relaxation in organizational contexts. Unlike prior research that has treated relaxation as a unidimensional construct or as a component of well-being (Sonnentag & Fritz, 2007), our multidimensional scale captures the complexity of relaxation as both a psychological and physiological state. The inclusion of a physical load dimension is particularly novel, aligning with recent calls to integrate embodied perspectives into organizational behavior research (Heaphy & Dutton, 2008).

Second, digital HRM as an antecedent of relaxation. Our finding that digital HRM positively predicts employee relaxation extends the growing literature on digital transformation in HRM (Strohmeier, 2020; Bondarouk & Brewster, 2016). Comprehensive reviews of electronic HRM have documented its adoption patterns and consequences, while others have highlighted how technology is fundamentally reshaping HRM functions (Stone et al., 2015). While previous research has focused on how digital HRM enhances efficiency and performance (Marler & Fisher, 2013), our study reveals a previously underexplored benefit: employee psychological well-being. This finding is particularly relevant given the increasing prevalence of technostress in the digital workplace (Tarafdar et al., 2007).

Third, the mediating role of work autonomy. Consistent with job demands-resources theory (Bakker & Demerouti, 2007), our results show that work autonomy serves as a key mechanism linking organizational resources (digital HRM) to individual outcomes (relaxation). This finding aligns with recent studies showing that autonomy is particularly valued by Generation Z employees (Scholz & Grotefend, 2019; Barhate & Dirani, 2022).

Fourth, the moderating role of digital self-efficacy. Our finding that digital self-efficacy strengthens the effect of digital HRM on work autonomy extends social cognitive theory (Bandura, 1997) to the digital HRM context. This suggests that digital HRM interventions may be more effective when combined with training programs that enhance employees’ digital confidence.

Our findings are consistent with several recent international studies. For example, Wu et al. (2025a) found that digital-intelligence transformation positively influences employee intentions in the workplace. Similarly, Jeong and Jeong (2025) demonstrated that self-efficacy mediates the relationship between AI-enhanced work environments and employee creativity. Our study complements these findings by identifying work autonomy as a mediating mechanism in the digital HRM–relaxation relationship.

### 6.3. Theoretical Contribution

Firstly, based on literature review and in-depth interviews, this study developed a standardized scale for measuring employees’ relaxation, encompassing four dimensions: work disengagement, work adaptation, emotional regulation, and physical load. This scale demonstrates good reliability and validity, serving as a localized measurement tool rooted in the Chinese organizational context. It provides a solid foundation for subsequent empirical research to explore the antecedents and consequences of employees’ relaxation.

Secondly, this study expands the research horizon of digital human resource management in terms of outcome variables. Existing research mostly focuses on the application of digital technology in human resource management and its conceptual connotation, lacking empirical examination of its relationship with employee attitudes and behaviors. Taking the relaxation of Generation Z employees as the entry point, this paper constructs a mechanism model with work autonomy as the mediator and digital self-efficacy as the moderator, revealing the internal path through which digital human resource management affects individual psychological states. This provides a new theoretical perspective for understanding its effectiveness boundary and application context.

Thirdly, this study advances COR theory in three important ways. First, while most COR research has focused on resource loss spirals and negative outcomes, we demonstrate a resource gain spiral in the context of digital HRM and Generation Z employees, showing how initial resource gains cascade into enhanced work autonomy and relaxation. Second, we identify work autonomy as a key mediating mechanism in the resource gain spiral, specifying how organizational resources translate into individual psychological relaxation. Third, by focusing on Generation Z, we extend COR theory to a new generational context, revealing that resource dynamics may differ across cohorts. Generation Z employees’ high digital literacy and strong need for autonomy make them particularly responsive to digitally enabled resource gains, suggesting that COR effects may be moderated by generational characteristics.

Finally, this study focuses on the Generation Z employee group with distinctive generational characteristics, systematically elaborating on the impact mechanism of digital human resource management on these employees’ sense of relaxation. The research findings deepen the understanding of the effectiveness of digital management practices in the context of generational differences and provide a theoretical basis for future research on generational comparison and differentiated human resource management.

### 6.4. Management Implications

Firstly, promote the construction of digital human resource management to create a flexible and efficient working environment. Generation Z employees are familiar with digital tools, so enterprises should establish a comprehensive digital human resource management system to provide a flexible, efficient, and personalized work experience. Enterprises can explore systems such as remote work and flexible working hours to meet their needs for work–life balance. They can utilize platforms like WeChat Work and DingTalk to establish cross-departmental communication groups, promote information sharing, enhance team collaboration efficiency, and create a relaxed atmosphere. At the same time, they can leverage digital platforms to provide rich online learning resources and personalized career development paths, helping them improve their skills and enhance their confidence in facing work challenges.

Secondly, grant more work autonomy to Generation Z employees. Work autonomy is a valued concept for Generation Z, and enterprises can empower them with more autonomy through digital human resource management, allowing them to independently arrange work schedules and choose work methods. This enhances their sense of control and responsibility, and improves relaxation and work efficiency. Enterprises can implement goal management, clarify goals and evaluation criteria, and allow employees to independently arrange their work. Encourage them to participate in decision-making processes such as project planning and workflow optimization, enhancing their sense of participation and belonging. On the other hand, an internal talent market can be established to provide opportunities for cross-departmental and cross-positional job rotations, allowing them to choose their development direction based on their interests and abilities, and to realize their self-worth.

Thirdly, focus on cultivating the digital self-efficacy of Generation Z employees. Enhancing their digital literacy helps them better adapt to and utilize digital human resources, and gain higher work autonomy and relaxation. Regular digital skills training courses can be organized to help them master the latest digital tools and technologies; establish a learning-oriented organization, encourage the sharing of learning experiences, and create a positive learning atmosphere; provide mobile office equipment such as tablet computers, and offer technical support such as online collaboration, to help them complete tasks efficiently and improve work efficiency.

### 6.5. Research Limitations and Prospects

Although this study pursues rigor in research design, it also has certain limitations. Firstly, it only explores the impact of digital human resource management at the individual employee level. Future research could conduct cross-level and multi-level studies, such as investigating the impact of organizational-level digital human resource management on employees’ individual-level cognition and emotions. Secondly, this study focuses on the positive impacts of digital human resource management on employees. Future research could explore the negative impacts of digital human resource management, such as issues related to privacy information leakage caused by digital human resource management or the double-edged sword effect it brings to employees. Thirdly, this study confirms the mediating role of work autonomy between digital human resource management and employee relaxation. However, there are other mediating variables between the two, such as job stress and perceived organizational support. Fourth, while we conceptualize relaxation as a positive low-strain state, some dimensions (e.g., work disengagement, physical load) may appear negative at first glance. We acknowledge that these dimensions capture the absence of strain rather than the presence of high-activation positive emotions. Additionally, this study relied exclusively on self-reported data for all variables. Although we took procedural precautions (e.g., time-lagged collection, anonymous responses), self-reports may still introduce same-source bias. Future research could employ multi-source designs (e.g., supervisor-rated autonomy, objective records) to complement self-reported data and further validate the conceptualization of relaxation by examining its nomological network with other positive and negative constructs.

## Figures and Tables

**Table 1:** Spiral coding of employee relaxation.

Main Category	Sub-Category	Initial Category
work disengagement	job detachment	Set work messages to do not disturb after work hours; establish clear boundaries for work tasks
	pressure infiltration degree	work pressure affects life emotions; work pressure leads to restlessness during rest time
	amount of private time	Sufficient time to spend with family; ample time for personal leisure activities
	regularity of daily routine	maintain a regular sleep schedule; avoid staying up late for overtime work; uphold healthy living habits
work adaptation	interpersonal harmony	supportive communication among colleagues; low-conflict interpersonal environment
	team harmony	the team cooperation atmosphere is relaxed and pleasant; a team atmosphere of mutual trust and mutual assistance
	environmental comfort	comfortable physical office environment; low-distraction workspace
	leadership affinity	leadership understanding and support; open and inclusive leadership style; timely and effective recognition and feedback
	work autonomy	flexibility in task arrangement; autonomy in choosing work methods; autonomy in arranging work hours
emotional regulation	emotional regulation ability	emotional self-awareness and adjustment; ability to maintain positive emotions
	challenge positivity	Embracing challenges proactively; maintaining an optimistic attitude in difficult situations
	adaptability to change	Quickly adapt to job changes; flexibly respond to uncertainties
	stress and anxiety	Anxiety during work; fear when facing work challenges
physical load	tense feeling in the body	I often feel tense or stiff during work; my muscles are highly tense during work
	fatigue accumulation degree	after work, I often feel physically exhausted; the accumulated level of work fatigue is high
	relax initiative	actively relieve physical fatigue through stretching; regularly arrange rest or engage in relaxing activities

**Table 2:** Exploratory factor analysis results.

Dimension	Item	Component	Factor 1	Factor 2	Factor 3	Factor 4	Commonality
work disengagement	A1	I can detach myself from work after getting off work	0.853				0.839
	A2	The pressure from work makes me prone to irritability and restlessness during my leisure time	0.788				0.823
	A3	In my life, I have ample time to spend with my family or pursue activities I enjoy	0.867				0.854
	A4	Even when I am busy with work, I can maintain a regular routine and healthy habits	0.816				0.826
work adaptation	B1	I have harmonious relationships with my leaders and colleagues		0.829			0.741
	B2	The atmosphere of my team collaboration is relaxed and pleasant		0.808			0.724
	B3	I am satisfied with the office environment		0.835			0.750
	B4	The way my leader gives feedback makes me feel respected		0.756			0.639
emotional regulation	C2	Demonstrate a positive attitude when facing work challenges			0.799		0.819
	C3	I can adapt to sudden changes in work			0.812		0.849
	C4	Exhibiting emotions such as anxiety and fear when confronted with work pressure or difficult problems			0.800		0.782
physical load	D1	I often feel tense or stiff in my body when I am working				0.869	0.821
	D2	After work, I often feel exhausted				0.884	0.854
	D3	I will actively alleviate physical fatigue through stretching or deep breathing				0.589	0.575
Eigenvalue (after rotation)			3.453	3.284	2.765	2.484	
Variance explained (%)			21.579	20.525	17.282	15.527	
Cumulative variance explained (%)			21.579	42.104	59.387	74.913	

**Table 3:** Results of confirmatory factor analysis.

Model	Factor	$\chi^{2}/df$	GFI	CFI	NFI	TLI	RMSEA	SRMR
Single-factor model	A+B+C+D	8.85	0.724	0.749	0.727	0.703	0.152	0.093
Two-factor model	A+B; C+D	7.97	0.733	0.78	0.757	0.736	0.143	0.096
Three-factor model 1	A+B; C; D	5.809	0.806	0.852	0.828	0.818	0.119	0.081
Three-factor model 2	A; B+C; D	5.473	0.815	0.863	0.838	0.831	0.115	0.07
Three-factor model 3	A; B; C+D	4.28	0.847	0.899	0.873	0.876	0.098	0.068
Four-factor model	A; B; C; D	1.951	0.945	0.972	0.945	0.945	0.053	0.042

**Note:** A: work disengagement; B: work adaptation; C: emotional regulation; D: physical load. “+”: factor combination.

**Table 4:** Test of discriminant validity of the scale.

Dimension	Work Disengagement	Work Adaptation	Emotional Regulation	Physical Load
work disengagement	(0.739)			
work adaptation	0.471 **	(0.792)		
emotional regulation	0.532 **	0.565 **	(0.829)	
physical workload	0.489 **	0.632 **	0.502 **	(0.728)

**Note:** ** *p* < 0.01.

**Table 5:** Results of confirmatory factor analysis.

Model	$\chi^{2}$	df	$\chi^{2}/df$	CFI	TLI	RMSEA	SRMR
Four-factor model (A, B, C, D)	991.5	521	1.903	0.953	0.950	0.050	0.041
Three-factor model (A, B+C, D)	1643.3	524	3.136	0.889	0.881	0.077	0.072
Two-factor model (A+B, C+D)	3018.3	526	5.738	0.753	0.736	0.114	0.128
Single-factor model (A+B+C+D)	4832.2	527	9.169	0.573	0.545	0.150	0.173

**Note.** A: digital human resource management; B: digital self-efficacy; C: work autonomy, D: employee relaxation; “+”: factor combination.

**Table 6:** Descriptive statistics and correlation analysis results.

Variable	1	2	3	4	5	6	7	8	9
1. gender	1								
2. age	−0.04	1							
3. education	−0.08	0.253 **	1						
4. years of work experience	−0.03	0.715 **	−0.10	1					
5. nature of the enterprise	0.05	−0.09	−0.07	−0.01	1				
6. digital human resource management	−0.01	0.12 *	0.006	0.05	−0.07	1			
7. work autonomy	−0.02	−0.01	−0.05	−0.02	−0.07	0.27 **	1		
8. digital self-efficacy	0.02	0.02	−0.03	0.01	−0.01	0.36 **	0.32 **	1	
9. employee relaxation	−0.06	0.12 *	0.05	0.07	−0.02	0.32 **	0.38 **	0.289 **	1
Mean	1.49	2.61	2.19	2.01	2.52	3.46	3.60	3.90	3.75
SD	0.50	1.12	0.78	0.90	0.78	0.94	1.36	1.50	1.27

**Note:** N = 364; * *p* < 0.05, ** *p* < 0.01.

**Table 7:** Hierarchical regression analysis results.

Variable	Work Autonomy				Employee Relaxation		
	M1	M2	M3	M4	M5	M6	M7
gender	−0.018	−0.019	−0.022	−0.018	−0.055	−0.055	−0.049
age	0.033	−0.039	−0.049	−0.041	0.130	0.068	0.080
academic qualification	−0.054	−0.033	−0.017	−0.019	0.006	0.024	0.034
years of work experience	−0.054	−0.020	−0.008	0.030	−0.296	0.004	0.011
nature of business	−0.071	−0.051	−0.051	−0.052	−0.173	0.008	0.024
digital human resource management		0.365 **	0.303 **	0.281 **		0.312 **	0.197 **
work autonomy							0.314 **
digital self-efficacy			0.233 **	0.225 **			
digital human resource management × digital self-efficacy				0.106 *			
$R^{2}$	0.008	0.138	0.188	0.199	0.017	0.112	0.197
$\Delta R^{2}$	0.008	0.130	0.050	0.010	0.017	0.095	0.085
F	0.584	9.561 **	11.806 **	11.005 **	1.256	7.513 **	12.482 **

**Note.** N = 364; * *p* < 0.05, ** *p* < 0.01.

**Table 8:** Test results of moderated mediation effect.

Variable	Digital Human Resource Management	Work Autonomy	Employees Relaxation	
	b	SE	CI (95%)	
low digital self-efficacy	0.079	0.032	0.021	0.149
high digital self-efficacy	0.160	0.040	0.086	0.242
difference	0.119	0.031	0.064	0.184

**Note.** b: unstandardized coefficient; SE: Standard Error; CI: Confidence Interval.

## Data Availability

The data presented in this study are available on request from the corresponding author due to privacy restrictions.
